# Retraction: Long non-coding RNA KCNQ1OT1 promotes osteosarcoma progression by increasing β-catenin activity

**DOI:** 10.1039/d1ra90013g

**Published:** 2021-01-20

**Authors:** Laura Fisher

**Affiliations:** Royal Society of Chemistry Thomas Graham House, Science Park, Milton Road Cambridge CB4 0WF UK advances-rsc@rsc.org

## Abstract

Retraction of ‘Long non-coding RNA KCNQ1OT1 promotes osteosarcoma progression by increasing β-catenin activity’ by Changsheng Zhang *et al.*, *RSC Adv.*, 2018, **8**, 37581-37589, DOI: 10.1039/C8RA07209D.

The Royal Society of Chemistry hereby wholly retracts this *RSC Advances* article due to concerns with the reliability of the data. The images in the article were screened by an image integrity expert. The expert found that the backgrounds to a number of western blot panels presented in Fig. 2I, 4E and 5G were identical and concluded that the bands had been added onto a false background.

The authors were asked to provide the raw data for this article, but did not respond. Given the significance of the concerns about the validity of the data, and the lack of raw data, the findings presented in this paper are not reliable.

The authors have been informed but have not responded to any correspondence regarding the retraction.

Signed: Laura Fisher, Executive Editor, *RSC Advances*

Date: 7^th^ January 2021

## Supplementary Material

